# Development and psychometric properties of a general cancer stigma scale

**DOI:** 10.1002/ijc.70314

**Published:** 2025-12-24

**Authors:** Stephen M. Kimani, Gita Suneja, Agatha Bula, Chaorong Wu, Abhilasha Khatri, Nehal Bakshi, Olivia R. Hanson, Aparna Mangadu, Mercy Tsidya, Melissa A. Stockton, Brandon A. Knettel, Melissa H. Watt

**Affiliations:** ^1^ Department of Medicine University of North Carolina at Chapel Hill Chapel Hill North Carolina USA; ^2^ Department of Radiation Oncology University of Utah Salt Lake City Utah USA; ^3^ UNC Project Malawi Lilongwe Malawi; ^4^ Division of Epidemiology University of Utah Salt Lake City Utah USA; ^5^ Honors College University of Utah Salt Lake City Utah USA; ^6^ School of Medicine University of Utah Salt Lake City Utah USA; ^7^ Department of Population Health Sciences University of Utah Salt Lake City Utah USA; ^8^ Department of Psychiatry University of Pennsylvania School of Medicine Philadelphia Pennsylvania USA; ^9^ Duke University School of Nursing; ^10^ Duke Global Health Institute Durham North Carolina USA

**Keywords:** cancer, global oncology, psychometric scale, stigma, survivorship

## Abstract

Cancer stigma negatively impacts diagnosis, quality of life, and survival outcomes, yet stigma reduction efforts are hindered by the lack of a valid and universal scale to measure cancer stigma. This study aimed to develop and validate a global cancer stigma scale, the Cross‐Cultural Oncology Measure for Perception and Awareness of Stigma Scale (COMPASS), which measures internalized, anticipated, and enacted stigma. The scale was developed using mixed methods in two phases: (1) feedback from experts and people with cancer on an initial pool of items, and (2) psychometric validation with people with cancer. Data were collected from two cancer centers in Salt Lake City, Utah, USA, and Lilongwe, Malawi. We examined the scale's initial psychometric properties, including item‐total correlation, reliability, factorial validity, and construct validity. Initial qualitative interviews informed the generation and refinement of 50 items, which were then administered to 209 individuals with cancer. Psychometric analysis reduced the scale to 28 items distributed across three stigma domains: anticipated (8 items), internalized (8 items), and enacted (12 items). The refined scale demonstrated satisfactory psychometric properties, with confirmatory factor analysis supporting a three‐factor model fit (CFI = 0.859, RMSEA = 0.112, SRMR = 0.063) and strong reliability (Cronbach's alpha: 0.92 to 0.97). Validity was confirmed through strong correlations with related constructs. COMPASS offers a reliable, valid, and cross‐culturally applicable tool to measure the magnitude and impact of cancer stigma globally and facilitate the evaluation of stigma reduction interventions.

## What's New?

Cancer‐related stigma can fuel anxiety and depression and lead to self‐isolation, delayed treatment, and decreased quality of life. The extent to which stigma impacts cancer patients, however, remains uncertain. This study applied mixed methods in high‐ and low‐income settings, with cohorts in the U.S. state of Utah and Lilongwe, Malawi, to explore a novel means of evaluating stigma. Through cultural adaptation, psychometric testing, and refinement, a 28‐item scale was developed. Items were distributed across three domains: anticipated, internalized, and enacted. The scale is a promising tool for measuring stigma's impact on cancer patients and evaluating progress in stigma reduction programs.

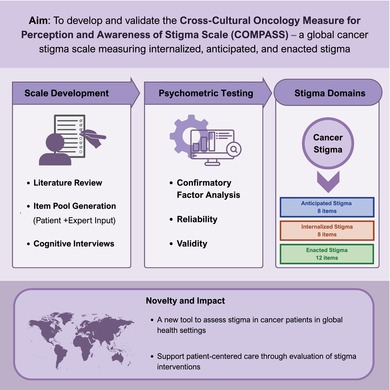

**Abbreviations**
CFA: Confirmatory factor analysis
CFI: Comparative fit index
COMPASS: Cross‐cultural Oncology Measure for Perception and Awareness of Stigma Scale
ECOG: Eastern Cooperative Oncology Group
FACIT: Functional Assessment of Chronic Illness Therapy
HCI: Huntsman Cancer Institute
IDIs: In‐depth interviews
KCH: Kamuzu Central Hospital
LMICs: Low‐ and Middle‐income countries
PROMIS®: Patient‐Reported Outcomes Measurement Information System
PTGI: Post‐Traumatic Growth Inventory
RMSEA: Root mean square error of approximation
SRMR: Standardized root mean square residual

## 1 BACKGROUND

Stigma is a physical or social attribute that devalues a person's identity, leading to exclusion and discrimination.^1^ Stigma encompasses an ‘*us versus them*’ ecosystem enabled by four conditions – power asymmetry, labeling, negative stereotyping, and separation– where people in a position to exercise power, label and assign stereotypes to others, and where such labeling encompasses negative attitudes or internalized beliefs about certain variations or characteristics that are considered deviations from societal norms.^2^, ^3^, ^4^ These characteristics could be physical, behavioral, or related to group membership. This leads to the separation of those labeled from others, creating a sense of “us” versus “them”. This social construct sets the stage for further stigmatization by distancing the labeled group from more desirable social groups, resulting in loss of status and various forms of discrimination. The power imbalance ensures that stigmatized groups remain at a disadvantage, creating a cycle that can be challenging to break.

Stigmatizing attitudes and practices are deeply embedded in societal norms and culture and extend to healthcare settings.^5^ Common drivers of stigma across disease conditions and populations include negative attitudes toward the patient population, fear of infection or contamination, association of the condition with unacceptable behaviors, and perceived discordance with cultural norms.^6^ Multidimensional measures of stigma have been developed and validated in relation to multiple chronic health conditions, including HIV,^7^, ^8^ mental health,^9^, ^10^ and epilepsy.^11^ Stigma related to cancer diagnosis encompasses negative societal attitudes or internalized beliefs directed towards individuals diagnosed with cancer, including experiences of discrimination, anticipation of prejudice, and acceptance of negative perceptions about oneself.^12^, ^13^ Stigma related to cancer diagnosis has direct and indirect effects on mental health. For instance, experiences with stigma related to cancer may increase the burden of anxiety and depressive symptoms, which may lead to social self‐isolation.^14^ Additionally, out of fear of discrimination, patients with cancer may choose to hide their conditions, leading to a lack of disclosure and isolation.^12^ In turn, social isolation frays social support, further exacerbating mental health distress through these interrelated cyclical patterns.^15^ Stigma serves as an impediment to engagement across the cancer care continuum, leading to delayed presentation, poorer treatment, elevated morbidity and mortality, and reduced quality of life.^16^, ^17^, ^18^ Cancer stigma appears to be more prevalent in low‐ and middle‐income countries (LMICs),^13^, ^19^, ^20^ where cancer care is limited, but observational studies are limited by the lack of a robust measurement tool that applies to all cancer types and geographical settings.

Patients with different types of cancer have different experiences of stigma and these experiences vary across different cultures and geographies given nuances in prevalence of risk factors, sociodemographic characteristics, physical manifestations of disease, and effect of disease‐specific interventions.^21^ For example, among breast cancer survivors, where mastectomy is often performed to treat breast cancer, women are more likely to experience stigma related to body image and perceptions of womanhood compared to men.^17^, ^22^ Similarly, while HPV is linked to other non‐cervical cancers (including anus and oropharyngeal), in communities where cervical cancer is perceived as associated with promiscuity, one may have stigma experiences unique to this diagnosis.^23^ As such, there is a role for cancer site‐specific stigma scales. Previous study has identified several useful cancer site‐specific scales. For cancers of the head and neck,^24^ lung,^25^, ^26^ breast,^27^ and rectum,^28^ measures of cancer stigma have been developed in high‐income countries, with some validation of lung cancer stigma scales in low‐ and middle‐income countries LMICs.^29^ A common denominator for these cancers with site‐specific stigma scales is that they are highly prevalent, have an identifiable lifestyle risk factor, or may be present with physical manifestations of the disease or treatment. However, there is pervasive prevalence of stigma related to carrying “cancer” as a diagnosis. Previous qualitative work identified general aspects of cancer‐related stigma, further lending credit to the idea of generalized cancer‐related stigma. Efforts to evaluate cancer‐related stigma reduction interventions are limited by the lack of a globally relevant scale that has been validated among populations with different types of cancer, with linguistic diversity, and from both high‐income and LMICs. There is need to develop a broader cancer stigma scale focusing on non‐attribution manifestations, that is, what patients “think, feel, and see”.

The goal of this study was to develop a Cross‐cultural Oncology Measure for Perception and Awareness of Stigma Scale (COMPASS) for global use, which includes three domains of stigma^30^: anticipated stigma (i.e., stigmatizing behavior someone anticipates or fears may happen to them), internalized stigma (i.e., negative, stigmatizing beliefs someone has internalized about themselves), and enacted stigma (i.e., stigmatizing behavior someone experienced) among cancer patients in Malawi and the United States using mixed‐methods approaches for scale development and validation.

## 2 METHODS

This study used a two‐step design and applied established mixed methods to develop and validate psychometric scales.^31^

### 2.1 Study setting

This study was conducted at two sites: Kamuzu Central Hospital (KCH) in Lilongwe, Malawi, and Huntsman Cancer Institute (HCI) at the University of Utah in Salt Lake City, Utah. KCH, Malawi's only tertiary hospital for ~9 million people in the central and northern regions, houses the Malawi National Cancer Center, offering comprehensive cancer care with government and partner support. HCI, the NCI‐designated Comprehensive Cancer Center for the Mountain West region of the USA (Utah, Idaho, Montana, Nevada, and Wyoming), serves five states spanning 17% of the continental US. Utah, home to HCI, has over three million residents, including a growing Latinx population (14%), one of the largest Pacific Islander populations in the continental US, a significant refugee community, and eight American Indian tribes/nations.

### 2.2 Participants

Perceptions of cancer and its associated stigmas can vary significantly across cultural contexts. In the US, there is often a lack of representation of racialized and minoritized groups in clinical research, leading to tools and interventions that may not fully address their specific needs or experiences.^32^ A comprehensive understanding of cancer stigma across different populations can provide valuable insights into universal versus context‐specific aspects of stigma. This justified our decision to develop and validate the scale centering the voices of people with cancer in Malawi and minoritized populations in Utah.

Oncology providers were eligible to participate as domain experts if they had at least 5 years of experience providing cancer care at either KCH or HCI. Patients were eligible to participate if they had received cancer treatment at either KCH or HCI and spoke Chichewa (KCH) or English (HCI). Patients at HCI were eligible to participate if they self‐identified as minoritized (American Indian/Alaska Native, Black/African American, Native Hawaiian/Pacific Islander, and Hispanic/Latinx).

### 2.3 Qualitative stigma measures development

#### 2.3.1 Item pool identification

Based on a review of existing stigma scales, we identified an initial pool of 50 items that reflected three domains of stigma: anticipated, internalized, and enacted. These items were selected based on established stigma theory,^33^ our qualitative work with cancer survivors in Malawi,^12^ and our work on developing HIV stigma measures in East Africa.^34^

#### 2.3.2 Assessment of content validity

We conducted in‐depth interviews (IDIs) with oncology providers at KCH (*n* = 6) and HCI (*n* = 6). The interview guide was organized into sections corresponding to the three stigma domains (anticipated, internalized, and enacted). For each domain, providers were asked to discuss how they saw this domain being expressed in their clinical practice. They then reviewed the item pool in each domain and provided feedback on the items, including the phrasing of items and whether to include or exclude them. Specifically, each provider was asked: (1) whether the set of items reflects their understanding of the domain, (2) whether any items were unclear or confusing or should not be included, and (3) whether additional items should be included.

#### 2.3.3 Translation and cross‐cultural adaptation and assessment of face validity

To assess the face validity of the item pool, we conducted cognitive interviews with eligible patients at KCH (*n* = 18) and HCI (*n* = 18). In Malawi, initial items were translated from English into *Chichewa* according to the Functional Assessment of Chronic Illness Therapy (FACIT) method to obtain cross‐cultural equivalence.^35^ FACIT translation methodology for cross‐cultural comparisons, which includes rigorous multistep processes, has been successfully applied to adapt health outcomes questionnaires into multiple languages. In our study, the English version of the item pool was forward translated into Chichewa by two independent bilingual native speakers of Chichewa. A third bilingual translator reconciled the two forward translations by selecting one forward translation or providing a new version of the translated items if there were differences. A fourth independent bilingual translator blindly back translated the reconciled version into English. Two independent bilingual experts reviewed the entire translation history and selected the most appropriate translation for each item or provided an alternate translation if previous translations were not acceptable. A bilingual investigator (Agatha Bula) assessed the recommendations of the expert reviewers to determine the finalized and harmonized item pools. Finally, trained bilingual qualitative interviewers used a semi‐structured interview guide to conduct cognitive interviews with eligible participants. Qualitative interviews were audio‐recorded, transcribed (and translated into Chichewa in Malawi), and iteratively analyzed through detailed summaries to inform subsequent revisions to the scale. All feedback was reviewed and discussed by the study staff (Stephen M. Kimani, Melissa H. Watt, Agatha Bula, and Mercy Tsidya) and upon consensus, items were revised, dropped, or added to capture the construct best. Each cognitive interview focused on a single domain of stigma (anticipated, internalized, and enacted) to obtain in‐depth feedback on items. Specifically, each patient was asked: (1) whether the set of items reflected the lived experiences of patients with cancer in their communities, (2) whether any items were unclear, confusing, or not relevant, (3) how they would respond to each item and why, in order to understand their use of the Likert scale response options, and (4) whether there were additional items that should be included to capture the lived experiences with the domain of stigma.

Qualitative interviews were audio‐recorded, transcribed (and translated from Chichewa in Malawi), and iteratively analyzed through detailed summaries to inform subsequent revisions to the scale. The study staff (Stephen M. Kimani, Melissa H. Watt, Agatha Bula, and Mercy Tsidya) reviewed all feedback and discussed to reach a consensus about revising, dropping, or adding items to capture the construct best.

### 2.4 Quantitative psychometric procedures

The pool of cancer stigma items was administered to patients receiving cancer treatment at the KCH (*n* = 100) and HCI (*n* = 109). The eligibility criteria for participation in the survey were the same as those for patient participation in the cognitive interviews (described above). For KCH, patients were approached in person during an oncology appointment, screened for eligibility, provided written informed consent, and then completed the survey using audio computer‐assisted self‐interview (ACASI) technology on an electronic tablet using the ODK Collect software program.^36^ For HCI, eligible patients were identified via electronic health records, and a letter with an individualized QR code was sent to complete the survey online using Research Electronic Data Capture (REDCap) hosted at the University of Utah.^37^

The pool of cancer stigma items administered to participants included 14 for anticipated stigma, 14 for internalized stigma, and 22 items for enacted stigma. The anticipated and internalized stigma domains had a 5‐point response set capturing the extent or magnitude of stigmatizing feelings or beliefs (0 = not at all, 1 = a little bit, 2 = somewhat, 3 = quite a bit, 4 = very much), while the enacted stigma domain had a 5‐point response set capturing the frequency of stigmatizing events (0 = never, 1 = rarely, 2 = sometimes, 3 = often, 4 = always).

In addition to the cancer stigma construct, the survey also included measures of demographics, mental health,^38^ social support,^39^ and resilency.^40^

### 2.5 Data analysis

#### 2.5.1 Preliminary analysis of item pool

We examined the response distribution of individual items through inspection of histograms (both combined and by country). Items with extreme skewness or kurtosis were excluded from subsequent analysis. Next, we examined item‐total correlations and Cronbach's alpha (combined and by country) for the three hypothesized dimensions of cancer stigma (anticipated, internalized, and enacted).

#### 2.5.2 Factorial validity

Confirmatory Factor Analysis (CFA) was performed using maximum likelihood estimation with robust standard errors (MLM) in Mplus version 8.10 (both combined and by country). Item reduction was carried out iteratively: investigators (Stephen M. Kimani, Melissa H. Watt and Chaorong Wu) reviewed the standardized loadings on the combined scale and group‐specific sample and identified items to be dropped based on: (1) loading below 0.7 and (2) theoretical discussion of the item's thematic content. A new CFA model was fitted after each round of item reduction, and this process continued until the model demonstrated a good fit and all standardized loadings exceeded 0.7, except for items deemed necessary for inclusion by the researchers.

We hypothesized a three‐factor model based on the latent constructs of internalized, anticipated, and enacted stigma. Model fit was evaluated using several indices: comparative fit index (CFI), root mean square error of approximation (RMSEA), and standardized root mean square residual (SRMR). Consistent with the best practices for fit indices, we considered CFI >0.85, RMSEA<0.06, and SRMR<0.08, to indicate a good or acceptable fit for this cross‐cultural measure.^41^, ^42^, ^43^

#### 2.5.3 Reliability

Scale reliability was analyzed separately for the three domains of cancer stigma (anticipated, internalized, and enacted), both combined and by country. Item‐total correlation and corrected item‐scale correlations (i.e., item‐total correlations with the other items in the scale, excluding the item itself) were reported. Cronbach's alpha coefficients were used to assess the internal consistency of the respective subscales. Coefficients between 0.7 and 0.9 were considered acceptable.

#### 2.5.4 Validity

We assessed the relationship between each stigma dimension and the domains of mental health (resiliency, depression, and anxiety) and social support (emotional, instrumental, and informational). Resiliency was assessed using the Post‐Traumatic Growth Inventory (PTGI) short form consisting of 10 questions with responses scored as follows: 0 = “not at all”, 1 = “a little bit”, 2 = “somewhat”, 3 = “quite a bit”, 4 = “very much”. PTGI is a tool to assess positive psychological changes following trauma. It measures growth in relationships, new possibilities, personal strength, spiritual change, and appreciation of life, underscoring the ability to recover or adapt to a previous level of emotional function after adversity.^40^ Depression, anxiety, and social support were assessed using their respective domain‐specific short forms adapted from the Patient‐Reported Outcomes Measurement Information System (PROMIS®). PROMIS® is a set of instruments developed by the NIH Common Fund initiative to standardize the measurement of key patient‐reported domains of well‐being.^44^ We hypothesized that participants with a higher endorsement of stigma would be more likely to endorse higher mental health distress (higher symptoms of anxiety and depression and lower resiliency) and lower social support. To support the scale validity, we considered a significant or trending association (*p* < 0.10) in the hypothesized direction. Measurement invariance was not conducted due to the small sample size across the two populations.^45^

## 3 RESULTS

### 3.1 Item development process and cross‐cultural adaptation

A 50‐item pool was identified from existing published measures. After expert review and cognitive debriefing with cancer patients, seven items were added and seven items were removed, leading to a pilot 50‐item scale that proceeded to psychometric testing (Table S1). Additionally, modifications were made to sentence structure and phrasing to improve understanding and cross‐cultural comprehension. Most participants understood the questions, were able to formulate accurate interpretations, and provided pertinent examples.

### 3.2 Characteristics of the sample for psychometric testing

In total, 209 participants responded to the item pool. Our sample was diverse in terms of race, ethnicity, sex, educational attainment, performance status, and duration of cancer survivorship (Table 1). We observed several significant demographic, clinical, and epidemiological differences between the patients in Malawi and Utah (Table 1). Compared with Utah, patients in Malawi were significantly younger (median age = 48.5 vs. 55.0 years, *p* = 0.01), with a higher proportion of males (54.0% vs. 38.5%, *p* = 0.03). While Black or African Americans were underrepresented in Utah (5.5%), our sample in Malawi was all Black (100%). In Utah, we enrolled a significantly diverse mix of races and ethnicities, with White Hispanics forming the majority (57%), and significant representation of Asian or Asian Americans (20%), American Indian/Alaska Native (9.2%), and Native Hawaiian/Other Pacific Islander (8.3%). There was a similar proportion of lymphoma cases (Malawi, 20.0%; Utah, 19.3%), but cervical cancer and Kaposi sarcoma were predominantly present in Malawi (21.0% and 14.0%, respectively), while leukemia and multiple myeloma were more common in the Utah cohort (17% and 7%, respectively). Most participants reported excellent performance status (56% with ECOG 0–1) and had been diagnosed with cancer more than 1 year before they participated in this study, with a median duration of cancer diagnosis of 4 years (interquartile range 3–7 years). Compared with Utah, a significantly higher proportion of patients in Malawi reported a shorter time (<1 year) since cancer diagnosis (61% vs. 30.3%, *p* < 0.01) and worse performance status (ECOG 2–3) (61% vs. 22.1%, *p* < 0.01).

**TABLE 1:** Participants' characteristics.

	Malawi (*N* = 100)	Utah (*N* = 109)	Overall (*N* = 209)	*p* value
	*n* (%)	*n* (%)	*n* (%)	
Gender				
Female	46 (46.0)	67 (61.5)	113 (54.1)	0.03
Male	54 (54.0)	42 (38.5)	96 (45.9)	
Race				
American Indian or Alaska Native	0 (0)	10 (9.2)	10 (4.8)	<0.01
Asian or Asian American	0 (0)	(20.2)	22 (10.5)	
Black or African American	100 (100.0)	6 (5.5)	106 (50.7)	
Native Hawaiian or Other Pacific Islander	0 (0)	9 (8.3)	9 (4.3)	
White^a^	0 (0)	62 (56.9)	62 (29.7)	
Ethnicity				
Hispanic or Latino	0 (0)	64 (58.7)	64 (30.6)	<0.01
Non‐Hispanic	100 (100.0)	45 (41.3)	145 (69.4)	
Age, years				
Median (IQR)	48.5 (38.8–57.2)	55.0 (43.0–64.5)	52.0 (40.0–61.0)	0.01
Education				
Less than high school	79 (79.0)	5 (4.6)	84 (40.2)	<0.01
High School	19 (19.0)	15 (13.8)	34 (16.3)	
Above high school	2 (2.0)	83 (76.1)	85 (40.7)	
Cancer type				
Lymphoma	20 (20.0)	21 (19.3)	41 (19.6)	NR
Breast cancer	9 (9.0)	14 (12.8)	23 (11.0)	
Cervical cancer	21 (21.0)	1 (0.9)	22 (10.5)	
Head and neck	5 (5.0)	8 (7.3)	13 (6.2)	
Gastrointestinal cancers	14 (14.0)	8 (7.3)	22 (10.5)	
Kaposi sarcoma	14 (14.0)	–	14 (6.7)	
Lung	3 (3.0)	1 (0.9)	4 (1.9)	
Prostate	4 (4.0)	4 (3.7)	8 (3.8)	
Leukemia	–	19 (17.4)	19 (9.1)	
Multiple myeloma	–	8 (7.3)	8 (3.8)	
Other^b^	10 (10.0)	19 (17.4)	29 (13.9)	
Performance status (ECOG)^c^				
ECOG 0	7 (7.0)	47 (43.1)	54 (40.7)	<0.01
ECOG 1	32 (32.0)	32 (29.4)	64 (30.6)	
ECOG 2	37 (37.0)	15 (13.8)	52 (24.9)	
ECOG 3	24 (24.0)	9 (8.3)	33 (15.8)	
Time since cancer diagnosis				
Less than 1 year	61 (61.0)	33 (30.3)	94 (45.0)	<0.01
More than 1 year	39 (39.0)	70 (64.2)	109 (52.2)	

*Note*: NR: *p* value not reported due to multiple categories.

^a^ In the US, race (i.e., skin color) and ethnicity (i.e., cultural identity) are captured as separate entities in the census report and included in healthcare delivery and research reports. People who identify as white (by race) and Hispanic (by ethnicity, based on cultural origin from Latin America) are considered culturally minoritized populations with unique healthcare access challenges.

^b^ Include uterine, ovarian, and melanoma.

^c^ ECOG 0–I am fully active and able to carry out activities the same as before my cancer diagnosis, without any restriction; ECOG 1–I have difficulty with physically strenuous activity, but I am able to walk and carry out work that is light or based in one location, such as light housework or office work; ECOG 2–I can walk and take care of myself, but I am not able to carry out work activities; I am up and about more than half the hours that I am awake; ECOG 3–I am capable only of limited self‐care and spend more than half the hours that I am awake in bed or in a chair.

### 3.3 Preliminary analysis of item pool

**FIGURE 1:**
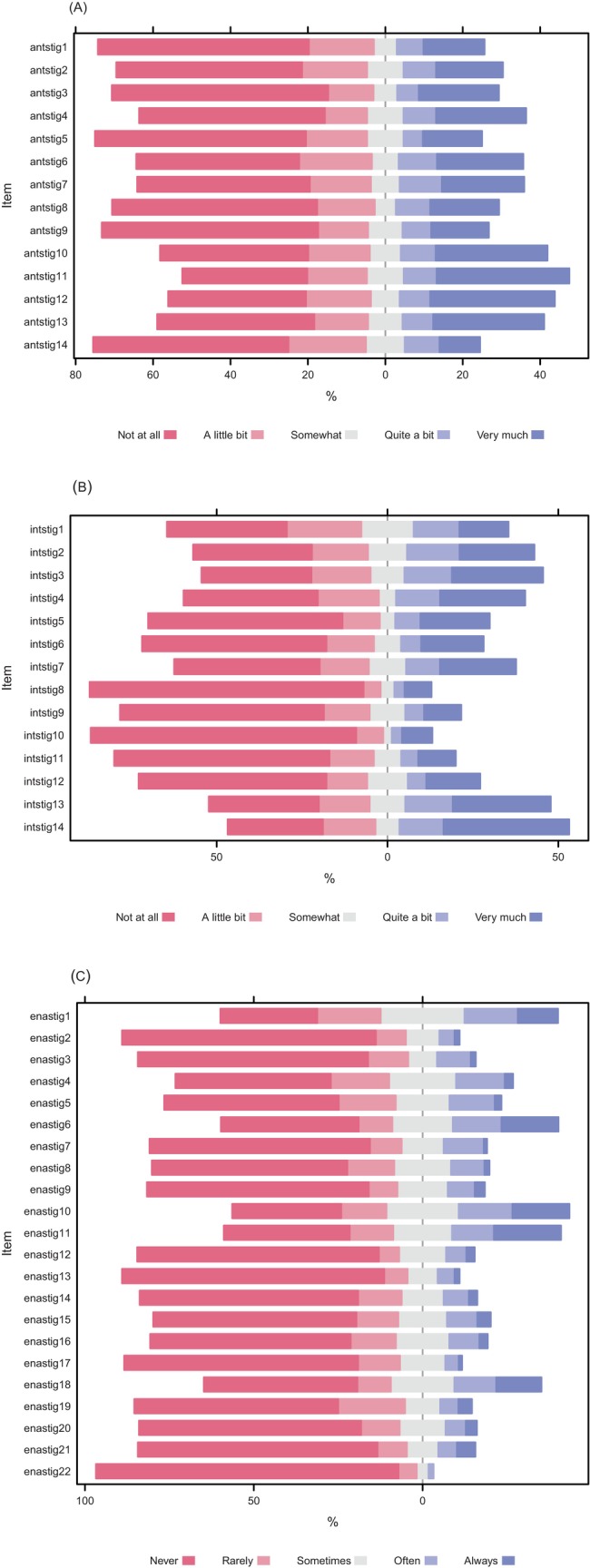
Stigma item endorsements for the initial pool of 50 items. (A) Anticipated stigma; (B) internalized stigma; and (C) enacted stigma. See positive endorsement proportions on the right side of the stacked bar chart and negative endorsement proportions on the left end.

The distribution of the 50 items was positively skewed (Figure 1). One item was removed because of an extremely skewed distribution in both samples (Enacted stigma, Item 22 “*I have been turned away from health care services outside of my cancer team because of my history of cancer*”). The remaining 49 cancer stigma items were included in the follow‐up analysis.

### 3.4 Factorial validity

**FIGURE 2:**
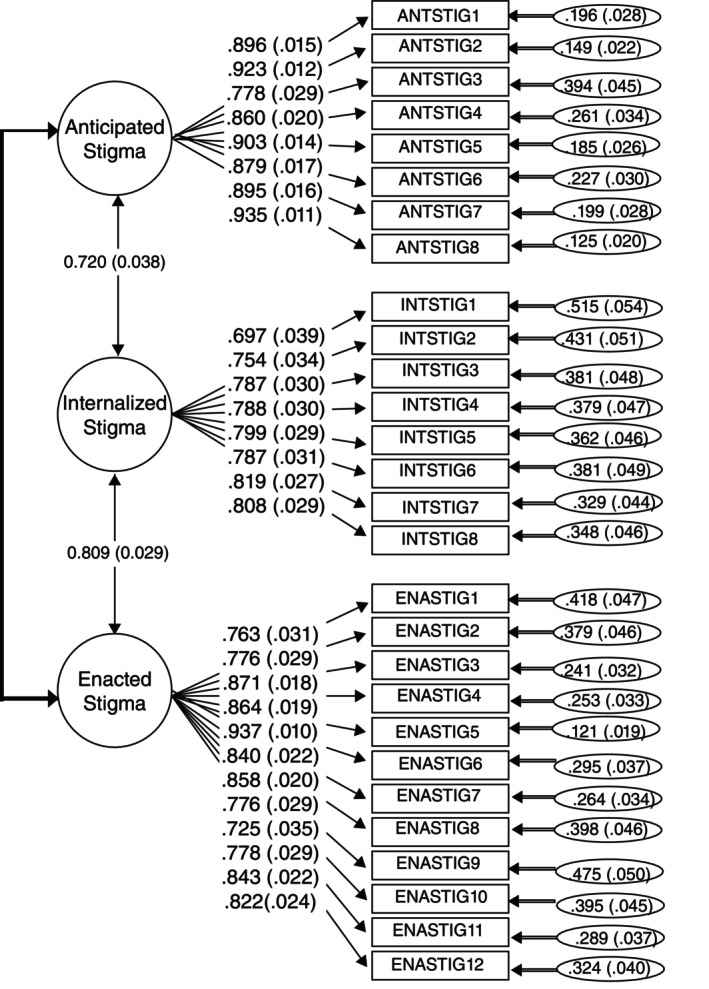
Final CFA model with standardized coefficients.

Confirmatory Factor Analysis was run iteratively with the combined sample for item reduction and factorial validity. In the final model, 21 items were dropped due to low item loadings (6 items in anticipated and internalized stigma each and 9 items in enacted stigma), leading to a final 28‐item scale (Table 2). As hypothesized, the three‐factor model showed an acceptable fit in the combined sample: χ^2^ = 1219.2, df = 347, *p* < 0.01; CFI = 0.859; RMSEA = 0.112 (90% CI: 0.105–0.118); and SRMR = 0.063. Moreover, the unstandardized factor loadings for all items were high and statistically significant, ranging from 0.71 to 1.29 (Table 3), with three distinct but correlated factors of cancer stigma (Figure 2).

**TABLE 2:** Summary of the 28‐item COMPASS scale with sources and modifications

Identifier	The final item in COMPASS scale	Original (Source)
Anticipated stigma		
ANTSTIG1	*I fear* people will be uncomfortable around me if they learn about *my cancer*.	Because of my illness, some people seemed uncomfortable with me (SSCI8^35^)^a^
ANTSTIG2	*I fear* people will avoid me if they learn about *my cancer*.	Because of my illness, people avoided looking at me (SSCI8^35^)
ANTSTIG3	I worry that people may judge me when they learn about *my cancer*.	I worry that people may judge me when they learn I have HIV (Berger stigma scale^36^)
ANTSTIG4	I worry about people *treating me badly* because of *my cancer*.	I worry about people discriminating against me due to my HIV (Berger stigma scale^36^)
ANTSTIG5	I fear some people will treat me like an inferior person because of my *cancer*	I feel some people treat me like an inferior person because of my epilepsy (Epilepsy scale^37^)
ANTSTIG6	*I fear* people will think that I am *going to die* if they learn about *my cancer*.	I felt that I did not deserve to live (HASI‐P^38^)^b^
ANTSTIG7	I worry about the way people will react to how my body has changed due to cancer.	New item
ANTSTIG8	I fear people will be uncomfortable around me because of my appearance due to cancer.	New item
Internalized stigma		
INTSTIG1	I feel *disconnected* from others because of my cancer.	I feel set apart from others who are well (Social impact scale^39^)
INTSTIG2	I am ashamed of *changes in my body since I was diagnosed with cancer*.	I am ashamed of my appearance (Shame and Stigma Scale^13^)
INTSTIG3	I feel ashamed for having developed cancer.	I feel ashamed for having developed cancer (Shame and Stigma Scale^13^)
INTSTIG4	I am embarrassed when *people learn that I have cancer*.	I am embarrassed when I tell people my diagnosis (Shame and Stigma Scale^13^)
INTSTIG5	I feel ashamed of my physical limitations *due to my cancer*.	I felt embarrassed because of my physical limitations (SSCI‐8^35^)^a^
INTSTIG6	Having *cancer* makes me feel like I'm a bad person.	Having HIV makes me feel I'm a bad person (Berger stigma scale^36^)
INTSTIG7	I feel I'm not as good as others because I have *cancer*.	I feel I'm not as good as others because I have HIV (Berger stigma scale^36^)
INTSTIG8	Having *cancer* makes me feel unclean.	Having HIV makes me feel unclean (Berger stigma scale^36^)
Enacted stigma		
ENASTIG1	I am treated differently because of *my cancer*.	In general, do you think people treat overweight people differently than normal weight people? (Prunty weight scale^40^)
ENASTIG2	Family members reject me because of *my cancer*.	Some family members have rehected me because of my illness (Social impact scale^39^)
ENASTIG3	Friends reject me because of *my cancer*.	I feel some friends have rejected me because of my illness (Social impact scale^39^)
ENASTIG4	Because of *my cancer*, others seem to feel uncomfortable when they are around me.	Because of my illness, some people seemed uncomfortable with me (SSCI8^35^)
ENASTIG5	Because of *my cancer*, people avoid me.	Because of my illness, some people avoided me (SSCI8^35^)
ENASTIG6	People avoid touching me if they know I have *cancer*.	People avoid touching me if they know I have HIV (Berger stigma scale^36^)
ENASTIG7	People I care about *have stopped communicating with me* after learning I have *cancer*.	People I care about stopped calling after learning I have HIV (Berger stigma scale^36^)
ENASTIG8	People act as though it was my fault that I have *cancer*.	Some people act as though it's my fault I have HIV (Berger stigma scale^36^)
ENASTIG9	People act like they are afraid they could “catch” *cancer from me*.	I feel others are concerned they could “catch” my illness through contact like a handshake or eating food I prepare (Social impact scale^39^)
ENASTIG10	People treat me like a child due to my cancer.	New item
ENASTIG11	People *treat me badly* because of *my cancer*.	People discriminate against me because I have a mental illness (ISMI^41^)^c^
ENASTIG12	*People* think I can't achieve *anything else* in my life because I have *cancer*.	Others think that I can't achieve much in life because I have a mental illness (ISMI^41^)

^a^ Stigma scale for chronic illnesses 8‐item version (SSCI8).

^b^ HIV/AIDS Stigma Instrument—PLWA (HASI‐P).

^c^ Internalized Stigma of Mental Illness (ISMI).

**TABLE 3:** Unstandardized factor loadings and corrected Item‐total correlation for the COMPASS scale.

	Item	Loading (*p* < 0.01)	Corrected item‐total correlation (CITC)
Anticipated stigma (8 items, Cronbach's alpha = 0.97)			
ANTSTIG1	I fear people will be uncomfortable around me if they learn about my cancer.	1.00	0.88
ANTSTIG2	I fear people will avoid me if they learn about my cancer.	1.03	0.91
ANTSTIG3	I worry that people may judge me when they learn about my cancer.	0.85	0.76
ANTSTIG4	I worry about people treating me badly because of my cancer.	0.89	0.84
ANTSTIG5	I worry about the way people will react when they learn about my cancer.	1.05	0.89
ANTSTIG6	I fear people will think that I am going to die if they learn about my cancer.	1.03	0.87
ANTSTIG7	I worry about the way people will react to how my body has changed due to cancer.	1.06	0.87
ANTSTIG8	I fear people will be uncomfortable around me because of my appearance due to cancer.	1.10	0.92
Internalized stigma (8 items, Cronbach's alpha = 0.92)			
INTSTIG1	I feel disconnected from others because of my cancer.	1.00	0.65
INTSTIG2	I am ashamed of changes in my body since I was diagnosed with cancer.	1.25	0.74
INTSTIG3	I feel ashamed for having developed cancer.	1.28	0.77
INTSTIG4	I am embarrassed when people learn that I have cancer.	1.23	0.76
INTSTIG5	I feel ashamed of my physical limitations due to my cancer.	1.29	0.78
INTSTIG6	Having cancer makes me feel like I'm a bad person.	0.95	0.73
INTSTIG7	I feel I'm not as good as others because I have cancer.	1.12	0.78
INTSTIG8	Having cancer makes me feel unclean.	1.00	0.75
Enacted stigma (12 items, Cronbach's alpha = 0.96)			
ENASTIG1	I am treated differently because of my cancer.	1.00	0.73
ENASTIG2	Family members reject me because of my cancer.	0.71	0.77
ENASTIG3	Friends reject me because of my cancer.	0.91	0.85
ENASTIG4	Because of my cancer, others seem to feel uncomfortable when they are around me.	1.01	0.84
ENASTIG5	Because of my cancer, people avoid me.	1.07	0.91
ENASTIG6	People avoid touching me if they know I have cancer.	0.92	0.83
ENASTIG7	People I care about have stopped communicating with me after learning I have cancer.	0.93	0.84
ENASTIG8	People act as though it was my fault that I have cancer.	0.86	0.76
ENASTIG9	People act like they are afraid they could “catch” cancer from me.	0.67	0.72
ENASTIG10	People treat me like a child due to my cancer.	0.86	0.76
ENASTIG11	People treat me badly because of my cancer.	0.76	0.84
ENASTIG12	People think I can't achieve anything else in my life because I have cancer.	1.18	0.80

### 3.5 Reliability

All three subscales demonstrated strong internal reliability: Cronbach's alpha was 0.97 for anticipated, 0.92 for internalized, and 0.96 for enacted stigma, respectively. The corrected item‐total correlations ranged from 0.65 to 0.92, with most items exhibiting strong correlations. Only one item [#1 “*I feel disconnected from others because of my cancer*.”] correlated lower than 0.70 (Table 3). However, because of its thematic importance, we retained this item on the final scale.

### 3.6 Validity

The included measures of resiliency, emotional distress, and social support demonstrated strong reliability in Malawi and Utah (Cronbach's alpha ≥0.90) (Table 4). All three subscales showed significant correlations in the expected direction with hypothesized correlates (Table 4). Associations with the emotional distress scales (PROMIS Depression and Anxiety Short Forms) were particularly strong. All three subscales showed correlations in the expected direction with measures of resilience and social support (Table 4).

**TABLE 4:** Spearman correlations of COMPASS scale and other measures.

Covariate	Scale	Group Cronbach's alpha		Stigma subscales		
		Malawi	Utah	Anticipated	Internalized	Enacted
Resiliency	PTGI^a^	0.91	0.91	−0.27***	−0.18**	−0.31***
Emotional distress	PROMIS**®** ^b^ Depression	0.92	0.92	0.68***	0.67***	0.76***
	PROMIS**®** Anxiety	0.95	0.92	0.56***	0.67***	0.69***
Social support	PROMIS**®** Emotional	0.96	0.94	−0.53***	−0.49***	−0.65***
	PROMIS**®** Instrumental	0.96	0.90	−0.34***	−0.42***	−0.54***
	PROMIS**®** Informational	0.95	0.94	−0.54***	−0.52***	−0.63***

*Note*: **p < 0.01, ***p < 0.001.

^a^ Post traumatic growth inventory (PTGI).

^b^ Patient‐reported outcomes measurement information system (PROMIS**®**).

### 3.7 Final Scale structure and scoring

Cross‐Cultural Oncology Measure for Perception and Awareness of Stigma Scale (COMPASS) is a 28‐item scale with three distinct subscales measuring anticipated, internalized, and enacted stigma domains (Data S1). Responses were rated on a 5‐point scale (0–4). The ratings for all items were summed to obtain a total score ranging from 0 to 112 for the combined domains. For each subscale, the total score ranges are as follows: anticipated (0–32), internalized (0–32), and enacted (0–48), with higher scores indicating greater levels of stigma.

## 4 DISCUSSION

We used a rigorous stakeholder‐engaged approach to develop a 28‐item psychometric scale, COMPASS, designed to measure anticipated, internalized, and enacted cancer stigma among adults in Malawi and the USA diagnosed with several different types of cancer. The results demonstrated that the COMPASS scale has strong psychometric properties, including content validity, internal consistency, and external validity. There were key demographic and clinical differences between the Malawi and USA cohorts, and the scale demonstrated utility in both settings.

As per the Health Stigma and Discrimination Framework,^6^ we hypothesized that inter‐ and intrapersonal stigma would cluster around three major domains: anticipated (the fear of experiencing discrimination or prejudice based on one's cancer diagnosis), internalized (individuals' personal negative beliefs and attitudes about cancer), and enacted (discriminatory behaviors and actions directed towards individuals affected by cancer). The factor loading in the confirmatory factor analysis was high and statistically significant, indicating that each item was strongly loaded onto its respective latent factor. Additionally, the fit indices suggest that the proposed model adequately fits the data. This confirms the scale's multidimensional nature, with three distinct but related subscales of cancer stigma: anticipated, internalized, and enacted.

The internal consistency of the scale, as measured by Cronbach's alpha, ranged from 0.92 to 0.97, indicating a high level of reliability. This is consistent with previous research on stigma measures in Malawi^9^, ^46^ and the USA,^25^ further supporting the robustness of the scale for measuring stigma in similar settings. Moreover, the scale showed good convergent validity through strong correlations with related constructs.^47^ Stigma scores across all three subscales were positively associated with the severity of emotional distress (i.e., depression and anxiety) and negatively associated with perceived social support. While stigma directly contributes to mental health distress, previous research has emphasized the adverse impact of stigma on social relationships, leading to isolation and loneliness, which further exacerbates mental health distress in a harmful cycle.^12^, ^14^, ^29^ Social support has consistently been shown to mitigate mental health distress^48^ through mechanisms such as reducing perceived stress and improving coping mechanisms.^49^ This protective role is particularly evident for informational and emotional support.^50^

The emergence of multiple stigma constructs in our analysis corroborates the work of Stangl et al.,^6^ who emphasized the complexity of the different stigma domains in their Health Stigma and Discrimination Framework. Their framework offers a comprehensive model for understanding and addressing health‐related stigmas, including those related to cancer, and is designed to inform interventions, policies, and research efforts by identifying the common domains and pathways of health‐related stigma. Moreover, this framework calls for ensuring the validity and reliability of stigma measures through rigorous psychometric testing and validation processes, which often involve piloting the instrument in diverse populations to assess its cultural relevance and appropriateness. Previous measures of cancer stigma have not adequately captured the unique sociocultural contexts and experiences of individuals in LMICs, where stigma may manifest differently owing to varying sociocultural norms, beliefs, and healthcare systems. While earlier work has focused on cancer‐specific forms of stigma,^17^, ^29^, ^51^ the development of the COMPASS tool advances the field by developing a validated tool for diverse cancer types and cross‐cultural applications in high‐ and low‐income settings, which has not previously been adequately addressed. The COMPASS can play a vital role in cancer stigma research and global health policy, including describing the prevalence and impact of stigma over place and time, evaluating the effectiveness of stigma reduction interventions, and producing evidence to inform policy advocacy and resource allocation.

The 20‐item COMPASS tool was developed as a self‐administered digital survey on available digital platforms in Malawi (audio‐assisted computer self‐interview) and Utah (web survey via REDCAP). We enrolled patients with cancer at various phases of care including those on active treatment and survivors on surveillance. As such, it has clinical applications such as psychosocial screening at diagnosis or follow‐up in oncology outpatient clinics or to assess ongoing stigma or identify supportive care needs in survivorship and palliative care programs. Moreover, it can be embedded in digital health tools such as eHealth and mHealth platforms for patient‐centered psychosocial health interventions. COMPASS can also be applied in oncology studies to assess the effect of stigma on outcomes such as quality of life, to measure effects of stigma‐reduction interventions in intervention trials, and to understand stigma experiences across demographic groups in regions where stigma impacts early diagnosis or care‐seeking behavior (e.g., rural or low‐resource settings). Further validation of this tool across larger and diverse settings is needed.

This study has several strengths, which contribute to its impact. First, we used a rigorous, mixed‐methods approach, ensuring that the COMPASS tool is theoretically grounded and robust. Including populations diverse in race, ethnicity, gender, educational attainment, performance status, and duration of cancer survivorship from two culturally distinct settings, the United States and Malawi, enhances the cross‐cultural relevance of the findings. Moreover, we enrolled individuals with a variety of cancer types, with the distribution reflecting the background prevalence in the respective regions, thus improving its applicability to global settings. Additionally, the scale demonstrates strong psychometric properties, with high reliability and validity across multiple stigma domains, positioning COMPASS as a valuable tool for assessing cancer stigma in global contexts.

Despite these strengths, this study had several limitations. First, our sample size was drawn from two tertiary cancer centers, potentially limiting the generalizability of the findings to other settings, such as non‐clinical settings. However, historically, the population of patients treated at the KCH and HCI has represented wide catchment areas spanning urban and rural communities, which may enhance the applicability of the findings to the respective regions. Our sample of two distinct populations had significant sociodemographic differences, reflecting unmeasured community‐level factors and the existing disparities in care. For instance, the US population had more cases of non‐lymphoma hematologic cancers, higher education levels, and skewed toward older and female patients. In contrast, the Malawi sample had more infection‐related cancers, lower education levels, and skewed younger and male patients. It is possible that this heterogeneity and small sample size impacted our model fit and should be considered when interpreting the results. Due to our small sample size, restricting enrolment to English‐speaking participants in the United States allowed this formative study to maintain semantic clarity and minimize misinterpretation across different languages. Though this approach could limit applicability to other populations in the United States, our participants came from varied socio‐demographic backgrounds (e.g., education, age, urban vs. rural), suggesting important applications in varied socioeconomic settings. Future studies with sufficiently large and linguistically representative samples are needed to test the scale's properties in each group and ensure adaptation to non‐English languages.

Further research is needed to validate COMPASS across other cultural contexts, cancer types, and additional settings. Additionally, we suggest that future research explores its utility in longitudinal studies evaluating stigma over time and the causal relationships between stigma and clinical outcomes. These efforts will refine the scale and expand its applicability in global cancer stigma research.

## AUTHOR CONTRIBUTIONS

**Stephen M. Kimani:** Conceptualization; investigation; funding acquisition; writing – original draft; methodology; visualization; writing – review and editing; formal analysis; project administration; data curation; supervision; resources. **Gita Suneja:** Conceptualization; funding acquisition; writing – review and editing; supervision; resources; methodology. **Agatha Bula:** Supervision; project administration; writing – review and editing. **Chaorong Wu:** Formal analysis; writing – review and editing. **Abhilasha Khatri:** Data curation; writing – review and editing. **Nehal Bakshi:** Writing – review and editing; data curation. **Olivia R. Hanson:** Data curation; software; writing – review and editing; methodology. **Aparna Mangadu:** Visualization; data curation; writing – review and editing. **Mercy Tsidya:** Methodology; data curation. **Melissa A. Stockton:** Methodology; writing – review and editing. **Brandon A. Knettel:** Methodology; validation; writing – review and editing. **Melissa H. Watt:** Conceptualization; investigation; funding acquisition; writing – original draft; methodology; validation; writing – review and editing; formal analysis; project administration; supervision; resources.

## FUNDING INFORMATION

Data collection, analysis, and manuscript preparation for this study were supported by the National Institutes of Health (3P30CA042014‐33S5 and 3U54CA254564‐02S1) and Ben B., and Iris M. Margolis Foundation (Melissa H. Watt). The contributions to manuscript writing were supported by grants from the National Institutes of Health (K01MH130226 to Melissa A. Stockton, K08CA228631 to Gita Suneja, and UM1TR004409 to Chaorong Wu through the University of Utah Translational Research: Implementation, Analysis, and Design). The content is the sole responsibility of the authors and does not necessarily represent the official views of the NIH.

## CONFLICT OF INTEREST STATEMENT

All authors report no conflicts of interest.

## ETHICS STATEMENT

The study was approved by the Institutional Review Board of the University of Utah (IRB number 00159133) and the Malawi National Health Science and Research Committee (IRB number 3052). All participants provided written informed consent and received appropriate incentives after the survey was completed according to local regulations.

## Supporting information


**DATA S1.** Cross‐cultural oncology measure for perception and awareness of stigma scale (COMPASS).
**TABLE S1.** Summary of the pilot item pool, sources, and modifications made to original items following qualitative interviews.

## Data Availability

De‐identified individual level data are available from the corresponding author upon request.
