# Supplementary material for: Development and psychometric properties of a general cancer stigma scale
Source: Int J Cancer. 2025 Dec 24;158(10):2747–59. doi: 10.1002/ijc.70314 (PMC12904632; doi:10.1002/ijc.70314)
Supplement: Supplementary file 1 — DATA S1. Cross‐cultural oncology measure for perception and awareness of stigma scale (COMPASS). TABLE S1. Summary of the pilot item pool, sources, and modifications made to original items following qualitative interviews. [file IJC-158-2747-s001.pdf]

Development and Psychometric Properties of a General Cancer Stigma Scale

Stephen M. Kimani
Gita Suneja
Agatha Bula
Chaorong Wu
Abhilasha Khatri
Nehal Bakshi
Olivia R. Hanson
Aparna Mangadu
Mercy Tsidya
Melissa A. Stockton
Brandon A. Knettel
Melissa H. Watt

Table of Contents

Supplement Table 1. Summary of the pilot item pool, sources, and modifications made to original items following qualitative interviews 2

Appendix 1. Cross-cultural Oncology Measure for Perception and Awareness of Stigma Scale (COMPASS)..9

Supplement Table 1. Summary of the pilot item pool, sources, and modifications made to original items following qualitative interviews

	Identifier	Original item in COMPASS	Source	Modification
	**ANTSTIG1**	I worry about rejection from my family because of my cancer.<br>Some family members have rejected me because of my illness	Social impact scale (Fife & Wright, 2000)	Modified "my illness" to "my cancer"; rephrased to be anticipatory
	**ANTSTIG2**	I worry about rejection from my friends because of my cancer.<br>I feel some friends have rejected me because of my illness	Social impact scale (Fife & Wright, 2000)	Modified "my illness" to "my cancer"; rephrased to be anticipatory
	**ANTSTIG3**	I worry about rejection at my place of work because of my cancer.	New item	New item added
	**ANTSTIG4**	I fear someone telling others about my cancer without my permission.<br>I fear someone telling others about my illness without my permission	Social impact scale (Fife & Wright, 2000)	Modified "my illness" to "my cancer"
	**ANTSTIG5**	I feel I need to keep my cancer a secret.<br>I feel I need to keep my illness a secret	Social impact scale (Fife & Wright, 2000)	Modified "my illness" to "my cancer"
	**ANTSTIG6**	I fear people will be uncomfortable around me if they learn I have cancer.<br>Lately.because of my illness, some people seemed uncomfortable with me	SSCI8 (Molina et al, 2013)	Modified "my illness" to "my cancer"; rephrased to be anticipatory
	**ANTSTIG7**	I fear people will avoid me if they learn I have cancer.<br>Lately…because of my illness, people avoided looking at me	SSCI8 (Molina et al, 2013)	Modified "my illness" to "my cancer"; rephrased to be anticipatory
	~~**ANTSTIG8**~~	~~I am very careful who I tell that I have cancer.~~<br>~~I am very careful whom I tell that I have HIV~~	Berger stigma scale	Changed HIV to cancer<br><br>In Malawi, this item was found to lack specificity. Investigators decided to drop it.
	**ANTSTIG9**	I worry that people may judge me when they learn I have cancer.	Berger stigma scale	Changed HIV to cancer

		I worry that people may judge me when they learn I have HIV		
	**ANTSTIG10**	I worry about people discriminating against me due to my cancer.<br>I worry about people discriminating against me due to my HIV	Berger stigma scale	Added "due to my cancer"<br><br>Changed to read "treating me badly because of my cancer" instead of "discriminating"
	**ANTSTIG11**	I worry about the way people will react when they learn I have cancer.<br>I worry about the way people will react when they learn I have epilepsy	Viteva epilepsy scale	"I have cancer" changed "about my cancer"
	**ANTSTIG12**	I fear people will think that I am going to die if they learn I have cancer<br>I felt that I did not deserve to live	Holzemer HASI-P	
	~~**ANTSTIG13**~~	~~I avoid getting close to people who don't have cancer to avoid rejection.~~	ISMI	Modified "mental illness" to "cancer"<br><br>Item found to be complex and with many negatives. Investigators decided to drop it.
	**ANTSTIG14**	I worry about the way people will react to how my appearance has changed due to cancer.	New item	New item added
	**ANTSTIG15**	I fear people will be uncomfortable around me because of my appearance due to cancer.	New item	New item added
	**ANTSTIG18**	I avoid spending time with people because of my cancer.	New item	New item added
	**INTSTIG1**	I feel set apart from others who do not have cancer.<br>I feel set apart from others who are well	Social impact scale (Fife & Wright, 2000)	"Set apart from others who do not have cancer" found to have mixed meanings i.e., being special. Changed to "disconnected from others because of my cancer"

	**INTSTIG2**	Due to my cancer, I feel like a burden in my relationships.<br>Due to my illness, I have a sense of being unequal in my relationships with others	Social impact scale (Fife & Wright, 2000)	Modified "my illness" to "my cancer"<br>Modified “being unequal” to “like a burden”
	~~**INTSTIG3**~~	~~I feel less competent than I did before my cancer.~~<br>~~Some people act as though I am less competent than usual~~	Social impact scale (Fife & Wright, 2000)	Modified "my illness" to "my cancer"<br>This item was found to lack specificity. Investigators decided to drop it.
	**INTSTIG4**	Due to my cancer, I sometimes feel useless.<br>Due to my illness, I sometimes feel useless	Social impact scale (Fife & Wright, 2000)	Modified "my illness" to "my cancer"<br><br>“useless” was found to be emotionally heavy/triggering. Replaced by “feel I have less to contribute to my community”
	**INTSTIG5**	I am ashamed of changes in my appearance since I was diagnosed with cancer.<br>I am ashamed of my appearance	Shame and Stigma Scale (Kissane, 2011)	Added "since I was diagnosed with cancer"
	**INTSTIG6**	I feel ashamed for having developed cancer.<br>I feel ashamed for having developed cancer	Shame and Stigma Scale (Kissane, 2011)	
	**INTSTIG7**	I am embarrassed when people learn that I have cancer.<br>I am embarrassed when I tell people my diagnosis	Shame and Stigma Scale (Kissane, 2011)	Changed "when I tell people” to "when people learn"
	**INTSTIG8**	I feel embarrassed because of my physical limitations due to my cancer.<br>Lately…I felt embarrased because of my physical limitations	SSCI8 (Molina et al, 2013)	Added "due to my cancer"<br><br>“embarrassed” replaced by “ashamed”
	**INTSTIG9**	Having cancer makes me feel like I'm a bad person.<br>Having HIV makes me feel I'm a bad person	Berger stigma scale	Changed "HIV" to "cancer"
	**INTSTIG10**	I feel I'm not as good as others because I have cancer.	Berger stigma scale	Changed "HIV" to "cancer"

		I feel I'm not as good as others because I have HIV		
	**INTSTIG11**	Having cancer makes me feel unclean.<br>Having HIV makes me feel unclean	Berger stigma scale	Changed "HIV" to "cancer"
	**INTSTIG12**	I worry that it is my fault that I have cancer.<br>How much do you feel..that you have HIV because you have done wrong behaviors?	Steward HIV related stigma	Changed "HIV" to "cancer"
	~~**INTSTIG13**~~	~~It is difficult to talk to other people about my cancer.~~<br>~~It is difficult to tell people about my HIV infection~~	Kalichman stigma scale	Changed "HIV infection" to "cancer"<br><br>This item was found to lack specificity. Investigators decided to drop it.
	~~**INTSTIG14**~~	~~I hide my cancer status from others.~~<br>~~I hide my HIV status from others~~	Kalichman stigma scale	Changed "HIV" to "cancer"<br><br>This item was found to lack specificity. Investigators decided to drop it.
	~~**INTSTIG15**~~	~~I feel that I can no longer contribute to society because I have cancer.~~<br>~~I sometimes feel worthless because ol am HIV positive~~	ISMI	Changed "mental illness" to "cancer"<br><br>This item was found to lack specificity. Investigators decided to drop it.
	**INTSTIG16**	I feel out of place in the world because I have cancer.<br>I feel out of place in the world because I have a mental illness.	ISMI	Changed "mental illness" to "cancer"<br><br>“out of placed in the world” was found to have mixed meanings. It was replaced with “a loss of identity”
	**INTSTIG17**	I feel people with cancer cannot live a good, rewarding life.<br>People with mental illness cannot live a good, rewarding life.	ISMI	Changed "mental illness" to "cancer"<br><br>“people with cancer” felt to be othering, and therefore replaced with “I can no longer”

	**INTSTIG18**	I feel having cancer has ruined my life.<br>Having a mental illness has spoiled my life	ISMI	Changed "mental illness" to "cancer"
	**ENASTIG1**	I am treated differently because of my cancer.<br>In general, do you think people treat overweight people differently than normal weight people?	Prunty weight scale	Changed "weight" to "cancer"
	**ENASTIG2**	Family members reject me because of my cancer.<br>Some family members have rehected me because of my illness	Social impact scale (Fife & Wright, 2000)	Modified "my illness" to "my cancer"
	**ENASTIG3**	Friends reject me because of my cancer.<br>I feel some friends have rejected me because of my illness	Social impact scale (Fife & Wright, 2000)	Modified "my illness" to "my cancer"
	**ENASTIG4**	Because of my cancer, others seem to feel uncomfortable when they are around me.<br>Lately…because of my illness, some people seemed uncomfortable with me	SSCI8 (Molina et al, 2013)	Modified "my illness" to "my cancer" and simplified wording
	**ENASTIG5**	Because of my cancer, people avoid me.<br>Lately…because of my illness, some people avoided me	SSCI8 (Molina et al, 2013)	Modified "my illness" to "my cancer"
	**ENASTIG6**	I have been hurt by how people react to learning I have cancer.<br>Hurt by how people reacted to learning I have HIV	Berger stigma scale	Changed "HIV" to "cancer"
	**ENASTIG7**	People avoid touching me if they know I have cancer.<br>People avoid touching me if they know I have HIV	Berger stigma scale	Changed "HIV" to "cancer"
	**ENASTIG8**	People I care about have stopped calling me after learning I have cancer.<br>People I care about stopped calling after learning I have HIV	Berger stigma scale	Changed "HIV" to "cancer"<br><br>“calling” changed to “reaching out”
	**ENASTIG9**	People act as though it was my fault that I have cancer.	Berger stigma scale	Changed "HIV" to "cancer"

		Some people act as though it's my fault I have HIV		
	**ENASTIG10**	People treat me differently because they think I am going to die.<br>I was refused treatment because I was told I was going to die anyway.	Holzemer HASI-P	Modified to be more about general population and not providers
	**ENASTIG11**	People give me less work opportunities because of my cancer.<br>My employer denied me opportunities.	Holzemer HASI-P	Replaced “work” with “opportunities to contribute” since some individuals may not be working.
	**ENASTIG12**	People think that my cancer is a punishment from God.<br>I was told that God is punishing me.	Holzemer HASI-P	
	**ENASTIG13**	People act like they are afraid they could "catch" cancer from me.<br>I feel others are concerned they could “catch” my illness through contact like a handshake or eating food I prepare	Social impact scale (Fife & Wright, 2000)	Modified "my illness" to "my cancer"
	**ENASTIG14**	I am shown less respect because of my cancer.<br>have you ever been treated less well than others or shown less respect because of your weight?	Prunty weight scale	Changed "weight" to "cancer"
	**ENASTIG15**	People laugh at me because of the way I look due to my cancer.	Viteva epilepsy scale	Change “laugh at me” to “make me feel embarrassed”
	~~**ENASTIG16**~~	~~Cancer has made me physically weak, frail, or ill which has caused me to experience stigma from others~~	New item	This item was found to be complex and hard to understand. Investigators decided to drop it.
	**ENASTIG17**	People treat me like a child due to my cancer.<br>People often patronize me, or treat me like a child, just because I have a mental illness.	ISMI	
	**ENASTIG18**	People discriminate against me because I have cancer.	ISMI	Changed "mental illness" to "cancer"

		People discriminate against me because I have a mental illness.		
	**ENASTIG19**	People think I can't achieve anything else in my life because I have cancer. Others think that I can't achieve much in life because I have a mental illness.	ISMI	Changed "mental illness" to "cancer"
	~~**ENASTIG20**~~	~~People ignore me or take me less seriously because I have cancer.~~ ~~People ignore me or take me less seriously just because I have a mental illness.~~	ISMI	Changed "mental illness" to "cancer"<br><br>This item was found to lack specificity. Investigators decided to drop it.
	**ENASTIG21**	People can see that I am physically unwell, which causes them to stigmatize me.	New item	"stigmatize" replaced by "treat me negatively" due to feedback from participants
	**ENASTIG22**	My partner avoids touching me because of my cancer.	New item	New item added
	**ENASTIG23**	My spouse rejects me due to my cancer.	New item	New item added
	**ENASTIG24**	I have been turned away from health care services outside of my cancer team because of my history of cancer.	New item	New item added

Appendix 1. Cross-cultural Oncology Measure for Perception and Awareness of Stigma Scale (COMPASS)

	**Please respond to each question by marking one box per row**					
	The following items are statements that some people with cancer may say they are worried or fearful about because of their cancer.					
	Please respond with how much you feel this way for yourself.	***Not at all***	***A little Bit***	***Somewhat***	***Quite a bit***	***Very much***
ANTSTIG1	I fear people will be uncomfortable around me if they learn about my cancer.	☐$_0$	☐$_1$	☐$_2$	☐$_3$	☐4
ANTSTIG2	I fear people will avoid me if they learn about my cancer.	☐$_0$	☐$_1$	☐$_2$	☐$_3$	☐4
ANTSTIG3	I worry that people may judge me when they learn about my cancer.	☐$_0$	☐$_1$	☐$_2$	☐$_3$	☐4
ANTSTIG4	I worry about people treating me badly because of my cancer.	☐$_0$	☐$_1$	☐$_2$	☐$_3$	☐4
ANTSTIG5	I worry about the way people will react when they learn about my cancer.	☐$_0$	☐$_1$	☐$_2$	☐$_3$	☐4
ANTSTIG6	I fear people will think that I am going to die if they learn about my cancer.	☐$_0$	☐$_1$	☐$_2$	☐$_3$	☐4
ANTSTIG7	I worry about the way people will react to how my body has changed due to cancer.	☐$_0$	☐$_1$	☐$_2$	☐$_3$	☐4
ANTSTIG8	I fear people will be uncomfortable around me because of my appearance due to cancer.	☐$_0$	☐$_1$	☐$_2$	☐$_3$	☐4
	The following items are statements that some people may say <u>they feel about themselves</u> because they have cancer.					
	Please respond with <u>how much</u> you feel this way for yourself.	***Not at all***	***A little Bit***	***Somewhat***	***Quite a bit***	***Very much***
INTSTIG1	I feel disconnected from others because of my cancer.	☐$_0$	☐$_1$	☐$_2$	☐$_3$	☐4
INTSTIG2	I am ashamed of changes in my body since I was diagnosed with cancer.	☐$_0$	☐$_1$	☐$_2$	☐$_3$	☐4
INTSTIG3	I feel ashamed for having developed cancer.	☐$_0$	☐$_1$	☐$_2$	☐$_3$	☐4
INTSTIG4	I am embarrassed when people learn that I have cancer.	☐$_0$	☐$_1$	☐$_2$	☐$_3$	☐4
INTSTIG5	I feel ashamed of my physical limitations due to my cancer.	☐$_0$	☐$_1$	☐$_2$	☐$_3$	☐4
INTSTIG6	Having cancer makes me feel like I'm a bad person.	☐$_0$	☐$_1$	☐$_2$	☐$_3$	☐4
INTSTIG7	I feel I'm not as good as others because I have cancer.	☐$_0$	☐$_1$	☐$_2$	☐$_3$	☐4
INTSTIG8	Having cancer makes me feel unclean.	☐$_0$	☐$_1$	☐$_2$	☐$_3$	☐4
	The following items are statements that some people <u>may have experienced</u> because they have cancer.					
	Please respond with <u>how often</u> this has happened for you.	***Never***	***Rarely***	***Sometimes***	***Often***	***Always***
ENASTIG1	I am treated differently because of my cancer.	☐$_0$	☐$_1$	☐$_2$	☐$_3$	☐4
ENASTIG2	Family members reject me because of my cancer.	☐$_0$	☐$_1$	☐$_2$	☐$_3$	☐4
ENASTIG3	Friends reject me because of my cancer.	☐$_0$	☐$_1$	☐$_2$	☐$_3$	☐4
ENASTIG4	Because of my cancer, others seem to feel uncomfortable when they are around me.	☐$_0$	☐$_1$	☐$_2$	☐$_3$	☐4
ENASTIG5	Because of my cancer, people avoid me.	☐$_0$	☐$_1$	☐$_2$	☐$_3$	☐4
ENASTIG6	People avoid touching me if they know I have cancer.	☐$_0$	☐$_1$	☐$_2$	☐$_3$	☐4
ENASTIG7	People I care about have stopped communicating with me after learning I have cancer.	☐$_0$	☐$_1$	☐$_2$	☐$_3$	☐4
ENASTIG8	People act as though it was my fault that I have cancer.	☐$_0$	☐$_1$	☐$_2$	☐$_3$	☐4

Cross-cultural Oncology Measure for Perception and Awareness of Stigma Scale (COMPASS)

ENASTIG9	People act like they are afraid they could "catch" cancer from me.	☐0	☐1	☐2	☐3	☐4
ENASTIG10	People treat me like a child due to my cancer.	☐0	☐1	☐2	☐3	☐4
ENASTIG11	People treat me badly because of my cancer.	☐0	☐1	☐2	☐3	☐4
ENASTIG12	People think I can't achieve anything else in my life because I have cancer.	☐0	☐1	☐2	☐3	☐4
